# Body Mass Index Trajectories during 6–18 Years Old and the Risk of Hypertension in Young Adult: A Longitudinal Study in Chinese Population

**DOI:** 10.1155/2021/6646868

**Published:** 2021-07-15

**Authors:** Haoyue Teng, Jia Hu, Wenxin Ge, Qiling Dai, Ji Liu, Chengqi Xiao, Jieyun Yin, Xiaoyan Zhu

**Affiliations:** ^1^Department of Epidemiology and Health Statistics, Medical College of Soochow University, Suzhou, China; ^2^Suzhou Center for Disease Prevention and Control, Suzhou, Jiangsu 215004, China; ^3^Institute of Suzhou Biobank, Suzhou, Jiangsu 215004, China

## Abstract

**Background:**

Overweight/obesity in childhood is suggested to increase the risk of hypertension later in life. We aimed to assess whether and how body mass index (BMI) trajectories during 6–18 years of age are associated with hypertension in young adulthood (18–37 years) in the Chinese population.

**Methods:**

Based on the China Health and Nutrition Survey (CHNS), a total of 1,872 participants who received ≥2 measurements of BMI during 6–18 years and had assessment of blood pressure (BP) in young adulthood were included. BMI trajectories were explored using latent class growth mixture models, and associations between identified trajectories with hypertension in young adulthood were examined by logistic regression analyses.

**Results:**

Five heterogeneous BMI trajectories were identified: the low slow-increasing (20.03%), low moderate-increasing (56.14%), low rapid-increasing (17.04%), moderate-increasing (3.63%), and elevated-decreasing (3.15%) groups. Compared with the low slow-increasing group, another three increasing groups had gradually elevated risk of hypertension, yielding maximally adjusted odds ratio (95% confidence interval) (OR (95% CI)) of 2.48 (1.39–4.42), 3.24 (1.66–6.31), and 3.28 (1.19–9.08), respectively, whereas the elevated-decreasing group reversed overweight/obesity to normal weight in childhood, rendering its association with hypertension as not statistically significant (OR (95% CI) = 2.74 (0.98–7.65)).

**Conclusion:**

Our study indicates that there are varied BMI trajectories from childhood to adulthood and that an elevated BMI trajectory during childhood is related with an increased risk of hypertension in young adulthood. In contrast, weight loss of children with high initial BMI may mitigate or reverse the risk. Our findings emphasize the importance of BMI continuous monitoring during early life.

## 1. Introduction

Hypertension is a global public health problem, affecting one-third of adults in the whole world [1]. It is also a leading cause of cardiovascular disease (CVD) and the global burden of disease [2]. In 2015, systolic blood pressure (SBP) of 140 mmHg and above accounted for 14% of the global deaths, with a loss of disability-adjusted life-years of 1.43 million person-years [3]. With the increasing trend of unhealthy lifestyles in modern society, the incidence of hypertension is expected to progressively rise and show a younger trend [1]. Some evidence noted that there is a gradually attenuated association between hypertension and risk of CVD and all-cause death with the increase in hypertension onset age [4–7]. Additionally, hypertension onset age under 35 years, but not above 45 years, is associated with a significantly increased risk of target end-organ damage, even after taking the influence of SBP into account [8]. The life expectancy of early-onset hypertension is longer, and the treatment and control rates are lower than those of late-onset hypertension, thus early-onset hypertension should not be overlooked [4–7]. Therefore, it was recommended to pay more attention to preventing hypertension in early adulthood, especially in the most populated country (China) [9].

A considerable number of studies have indicated that adult hypertension originates in childhood [10, 11]. Overweight/obesity in early life is a well-acknowledged risk factor for hypertension [12, 13]. Childhood overweight/obesity is often accompanied by disturbances in autonomic function, insulin resistance, and abnormalities in vascular structure and function, which are closely involved in the development of hypertension [12–14]. In China, a national survey reported that the prevalence of overweight and obesity in children aged 7–17 years had increased rapidly from 3.8% and 0.6% in 1995 to 14.3% and 4.1% in 2014, respectively [15].

Body massive index (BMI) is widely accepted to assess children's weight status. It should be noted that BMI changes rapidly in childhood [16]. Recent evidence has suggested that there are varied BMI changing patterns among children, which may bring different health outcomes [17–19]. BMI measurements in the majority of existed epidemiological studies were just be performed at one or limited occasions [20, 21], which may be insufficient to explain the risk of hypertension. Traditional regression or growth curve modeling assumes only one mean within the population. In contrast, the latent class growth mixture (LCGM) model is an emerging method, the primary purpose of which is to identify groups of individuals with similar development trajectories [22]. A body of studies have successfully performed the LCGM model to identify BMI trajectories and provided more information about BMI and disease [19, 23, 24].

Given the urgent need to prevent hypertension in young adults, the alarming popularity of childhood overweight and obesity in China [15], and the well-document relation between childhood BMI and adult hypertension, it is necessary to explore the role of BMI trajectories during early life on the progression of hypertension in young adults. Therefore, based on the China Health and Nutrition Survey (CHNS) cohort study, we used the LCGM model to describe the BMI trajectories during 6–18 years of age in Chinese pediatric population and to investigate the relationship between identified trajectories and young adulthood hypertension.

## 2. Methods

### 2.1. Study Population

The current study is based on surveys of the CHNS that conducted in 1989, 1991, 1993, 1997, 2000, 2004, 2006, 2009, 2011, and 2015, with participants of each wave being followed up overtime [25, 26]. In brief, the survey uses a multistage, random cluster process to draw the surveyed samples from each province. Counties were stratified by income (low, middle, and high), and a weighted sampling scheme was used to randomly select four counties in each province. Villages and townships within the counties and urban/suburban neighborhoods within the cities were selected randomly. The primary sampling units have increased to 360—60 urban neighborhoods, 60 suburban neighborhoods, 30 towns, and 180 villages.

A total of 21,378 participants who were followed up between 6 and 37 years of age were extracted from the CHNS. Later, 1,108 and 669 participants without BMI or blood pressure (BP) values were excluded, respectively. Afterward, exclusions were subsequently made for 14,610 participants who received <2 measurements of BMI during 6–18 years of age and 3,119 participants who did not have BP assessment during young adulthood (18–37 years). Finally, a total of 1,872 participants were included in the present analysis.

**Figure 1:**
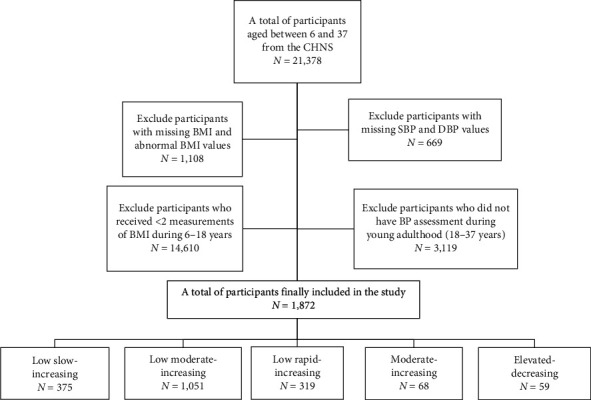
Flow chart for selection process of the study.

The flowchart of the exclusion and inclusion process of our study population is presented in Figure 1. The CHNS was approved by the institutional review committees of the Chinese Center for Disease Control and Prevention and the University of North Carolina in Chapel Hill. Written informed consent was obtained from children and their guardians during childhood, and participants' informed consent was obtained in early adulthood.

### 2.2. Data Collection

Height and weight in both childhood and early adulthood were measured without shoes and in light clothes. By using a calibrated beam scale, weight was measured to the nearest 0.1 kg in light clothing. Height was measured to the nearest 0.1 cm without shoes by using a portable stadiometer. BMI was calculated as weight in kilograms divided by the square of height in meters. Each adult's SBP and diastolic BP (DBP) were measured on the right arm with the lower edge of the cuff placed ∼25 mm above the elbow after a 10-min seated rest using mercury sphygmomanometers. When measuring BP in childhood, the width of the cuff inflatable bag is at least 40% of the circumference of the upper arm between the olecranon and the acromion, and the length is 80% to 100% of the circumference of the upper arm. The arm is bare, and the cuff of the sphygmomanometer is at the same level with the heart. SBP is recorded when the first time there is a clear flapping sound (Korotkoff Phase I). DBP is recorded when Korotkoff disappears (Korotkoff V period). BP was measured three times at 30-s intervals, and the mean of the three measurements was recorded.

### 2.3. Definitions of Terms

In childhood, the definition of overweight/obesity adopts the reference standards of the Centers for Disease Control and Prevention [27]. Overweight is defined as a BMI between the 85th and 95th percentiles of children and adolescents with the same age and sex, whereas obesity is defined as a BMI at or above the 95th percentile. In early adulthood, hypertension was defined as self-reported hypertension, SBP ≥ 140 mmHg or DBP ≥ 90 mmHg, or using antihypertension medications [28]. According to the standards of the Chinese Adult Overweight and Obesity Prevention and Control Guidelines, 24 kg/m^2^ ≤ BMI < 28 kg/m^2^ is considered as overweight, and BMI ≥ 28 kg/m^2^ is defined as obesity [29].

### 2.4. Statistical Analysis

#### 2.4.1. Identification of BMI Trajectories

The LCGM model was used to identify different trajectory patterns of BMI during 6–18 years of age. Due to the continuity of BMI, the censored conventional model was considered appropriate. We firstly compared the Bayesian Information Criterion (BIC) among the model with two to six trajectories, with all functional forms set to a third-order (cubic) equation. As recommended in the literature, the closer the BIC values to zero, the better the model is fitted [30]. When the two models are compared, the BIC log Bayes factor greater than 2 indicates that the model with a smaller absolute value of BIC fits better [31]. For each model involving latent classes, posterior class-membership probabilities were used to obtain a posterior classification of the participants in each latent class to evaluate goodness of fit and characterize the discrimination of latent clusters. We further retrieved the proportion of subjects classified in each class with a posterior probability above a threshold of 0.7, indicating the proportion of subjects unambiguously classified in each latent class [30]. The parameter estimates of model with 2 to 6 trajectories are given in Table 1. Thus, five trajectories were selected as the optimum model because of relatively small BIC values, high mean posterior probability, and percent of individuals with posterior probability >0.70. Furthermore, cubic, quadratic, and linear terms were evaluated based on their statistical significance after starting with the highest polynomial. In our final model, all of the five trajectories had cubic order terms. A flow chart of optimal model selection is present in Supplementary Figure 1.

#### 2.4.2. Basic Analyses

Differences in characteristics of the participants according to the five BMI trajectories were tested using the rank-sum test or 1-way ANOVA for continuous variables and the chi-square test for categorical variables, respectively. We calculated odds ratio (OR) (95% confidence levels (95% CIs)) in three logistic models to evaluate the associations between BMI trajectories and risk of hypertension in young adults. Model 1 was unadjusted. Model 2 adjusted for demographic characteristics of the subjects (sex, education level, and age at the last BP measurement). To control the possible influence of childhood hypertension status, which was not assessed because BP was only measured at single time point within 1 year in the CHNS, Model 3 additionally controlled for mean values of SBP and DBP (mmHg, continuous) during childhood based on Model 2. All statistical tests were performed using SAS software (version 9.4; SAS Institute, Cary, NC, USA), and differences were considered statistically significant when two-sided *P* ≤ 0.05.

## 3. Results

### 3.1. BMI Trajectories

**Figure 2:**
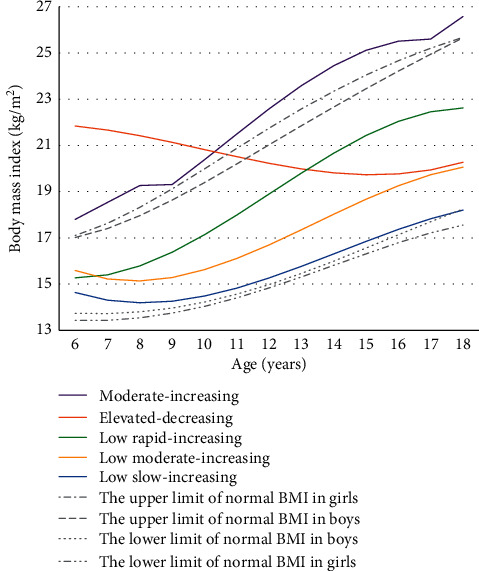
BMI trajectories from 6 to 18 years.

As shown in Figure 2, 1,872 participants were assigned into five different BMI trajectories from 6 to 18 years of age. According to the CDC growth reference data [27], overweight was defined as 16.8 ≤ BMI < 18.5 kg/m^2^ for boys and 17.0 ≤ BMI < 19.2 kg/m^2^ for girls at 6 years old. Therefore, we named low, moderate, and elevated BMI trajectories referring to normal weight, overweight, and obesity at the age of six, respectively. The numbers of participants were 375 (20.03%), 1,051 (56.14%), 319 (17.04%), 59 (3.15%), and 68 (3.63%) for the low slow-increasing, low moderate-increasing, low rapid-increasing, elevated-decreasing, and moderate-increasing groups, respectively.

The low slow-increasing group approximately follows the lower range of normal BMI [27], so we choose this trajectory as a reference. Both the low moderate-increasing and low rapid-increasing trajectories develop within the normal BMI range during childhood. The elevated-decreasing group has excessive BMI at initial and then gradually falls back into the normal range. The moderate-increasing group goes above the upper range of normal BMI throughout childhood.

### 3.2. Baseline Characteristics

The study variables by BMI trajectories are presented in Table 2. A total of 1,872 participants (males: 59.24%) with 7,703 BMI assessments in childhood (6–18 years old) were included. During childhood, the average BMI was 17.30 ± 2.90 kg/m^2^, and 89 (4.75%) and 69 (3.69%) subjects experienced or developed overweight or obesity, respectively. In early adulthood (18–37 years old), 162 (8.65%) participants suffered hypertension. Significant differences in characteristics (i.e., age, BMI, SBP, and DBP) at enrollment of the current study among the five trajectory patterns were observed (*P* < 0.001).

### 3.3. Associations of BMI Trajectories with Hypertension in Young Adulthood

As shown in Table 2, 4.53%, 9.42%, 10.97%, 8.47%, and 8.82% of participants were defined as hypertension in the low slow-increasing, low moderate-increasing, low rapid-increasing, elevated-decreasing, and moderate-increasing groups, respectively. Compared with the lowest trajectory (the low slow-increasing group), another three upward trajectories (low moderate-increasing, low rapid-increasing, and moderate-increasing groups) had elevated risk of hypertension during young adulthood, yielding maximally adjusted OR (95% CI) of 2.48 (1.39–4.42), 3.24 (1.66–6.31), and 3.28 (1.19–9.08), respectively. However, the association between the elevated-decreasing group and adult hypertension were neutral (OR (95% CI) = 2.74 (0.98–7.65)) (Table 3).

## 4. Discussion

In this longitudinal study, with 7,703 BMI measurements from 1,872 Chinese children and adolescents, we identified five distinct BMI trajectories from ages 6 to 18 years. We estimated the relationship between participants on different BMI trajectories and the incidence of hypertension in young adulthood and found that compared with the lowest BMI trajectory, upward trajectories were prone to hypertension. However, participants who remitted overweight/obesity to normal weight showed a neutral association with hypertension in early adulthood.

Studies have reported that an excessive increase in BMI in childhood can lead to hypertension in adulthood [30, 32, 33]. Similarly, we found that participants in the moderate-increasing group showed a more than 3-fold increased risk of hypertension compared with those in the low slow-increasing group, which suggests the importance of early intervention to prevent excessive weight gain in childhood. Notably, the other two increasing BMI trajectory groups in our study, despite within the normal range of BMI in childhood, showed an increased probability of hypertension. In particular, OR of hypertension for participants in the low rapid-increasing group was just slightly lower than that of the moderate-increasing group, indicating that although within the normal range, a rapid increase in BMI in early life may be unfavorable.

Besides these ascending trajectories, we also found a descending trajectory that was with neutral association with hypertension. Consistent with our findings, previous cohort studies have suggested that overweight/obesity in childhood but normal weight in early adulthood could alleviate the risk of adult hypertension in blacks, Hispanics, or white non-Hispanics [34, 35]. Another cohort study from China also showed that participants who transitioned from overweight in adolescence to normal weight in early adulthood had the same risk of hypertension as those who remained of normal weight from adolescence to adulthood [18]. These findings suggested that the influence of early childhood overweight/obesity on adult hypertension may be reversible and returning to normal weight in early adulthood may reduce the risk of hypertension in adulthood.

Meanwhile, it was found that the low moderate-increasing and elevated-decreasing BMI trajectories almost converged around 18 years of age in the current study. Interestingly, the two groups possessed opposite directed trajectories during childhood and led to varied probability of hypertension in young adulthood. This also suggested that it may be insufficient to assess disease risk only by BMI at one single point.

Potential mechanisms linking overweight/obesity to hypertension include hemodynamic changes, sympathetic nervous system activation, sodium retention, renal insufficiency, endocrine changes, insulin resistance and vascular dysfunction due to inflammation, and dietary factors [14, 36]. High-salt diet is positively associated with overweight/obesity independent of energy intake [37] and also a well-acknowledged risk factor for hypertension [38]. Reduction in salt intake was reported to lower BP among children and adolescents [39]. Therefore, it is highly recommended that children and adolescents should develop good eating habits and limit sodium intake. Meanwhile, physical examinations of children and adolescents should be regularly performed and the BMI trajectory could be determined. Especially, children and adolescents who had a persistent high BMI or a rapid increase in BMI during childhood should be closely monitored and given timely interventions [40].

Our data were derived from the CHNS cohort study, which is a longitudinal study with subjects from urban and rural areas in multiple provinces in China. It used standardized protocols and rigid quality control procedures, which may provide a valid appraisal of the relationship between BMI trajectories and the risk of hypertension. Nevertheless, there are some limitations of our study that should be considered. First, our cohort only includes Chinese. The BMI trajectory groups identified may not be generalizable to other populations. Second, the number of participants eventually included in the study is relatively small, and studies with larger sample size are needed to verify our results. Third, hypertension is suggested to be evaluated based on BP values from at least three separate office visits for both adults and children. However, BP was only measured at single time point within 1 year in the CHNS, and the status of hypertension for children and adolescents was not assessed. Therefore, our study was unable to exclude children and adolescents with hypertension at baseline. We tried to adjust the mean BP values during childhood as a compensation, which may not be sufficient to control their impact. Fourth, BMI does not necessarily reflect body fat changes and muscle mass that occur with age [41]. Fifth, potential confounding factors, such as early-life factors (e.g., birth weight and gestation age), family history of hypertension, nutrition factors (e.g., salt intake), and lifestyle factors (e.g., physical activity and sleep duration), were unavailable and were not adjusted in the current analyses.

## 5. Conclusions

Our research supports an association between the excess gain in BMI during childhood and increased chances of hypertension in young adulthood. In contrast, participants who reversed childhood overweight/obesity to normal weight in adolescents may mitigate the risk of hypertension. Such findings are of particular interest because childhood, a phase of very rapid growth, constitutes a critical period for the onset of overweight/obesity. Continuous BMI monitoring is essential in identifying high-risk children, and specific public policies and clinical attention should be formulated for this population. Additional research is needed to clarify childhood growth trajectories and their association with adult hypertension, to more effectively prevent overweight/obesity in children and hypertension in adults.

## Figures and Tables

**Table 1:** Parameter estimates for the latent class growth mixture modeling.

Nb. latent classes	Polynomial degree	Log-likelihood	BIC	% participants per class	Mean posterior probabilities	Posterior probabilities >0.7 (%)
2	Cubic	−10461.73	−10499.40	83.55/16.45	0.97/0.87	96.68/78.57
3	Cubic	−10323.23	−10379.74	65.38/27.83/6.78	0.88/0.80/0.88	85.78/72.55/83.46
4	Cubic	−10196.91	−10272.25	64.53/27.30/4.70/3.47	0.88/0.89/0.80/0.84	85.43/85.23/69.47/75.38
5	Cubic	−10147.65	−10241.83	20.03/56.14/17.04/3.15/3.63	0.75/0.79/0.76/0.83/0.88	61.90/70.89/68.72/76.39/83.33
6	Cubic	−10075.40	−10188.42	19.18/55.07/17.68/2.83/1.44/3.79	0.80/0.79/0.77/0.76/0.89/0.88	64.15/70.39/64.90/68.77/81.48/80.28

Parameter estimates include the number of latent classes considered, the polynomial form of the model, the maximum log-likelihood, and the Bayesian Information Criterion (BIC), and for models with 2 or more classes, the a-posteriori classification of subjects in each class (%), the mean of posterior probabilities in each latent class, and the % of subjects classified in each class with a posterior probability above 0.7 are considered.

**Table 2:** Characteristics of study participants in childhood and early adulthood.

Variables, *n* (%), mean (SD) or median (interquartile range)	Total population (*n* = 1872)	Low slow-increasing (*n* = 375)	Low moderate-increasing (*n* = 1051)	Low rapid-increasing (*n* = 319)	Moderate-increasing (*n* = 68)	Elevated-decreasing (*n* = 59)	*P* value
*Childhood*							
Males (*n*, %)	1109 (59.24%)	235 (62.67%)	627 (59.66%)	170 (53.29%)	45 (66.18%)	32 (54.24%)	0.0737
Age^*∗*^ (years)	11.52 ± 3.13	11.62 ± 3.03	11.46 ± 3.23	11.60 ± 3.16	11.78 ± 3.09	9.51 ± 1.70	<0.0001
Height (cm)	139.52 ± 16.91	138.23 ± 16.80	139.84 ± 16.73	140.94 ± 17.45	144.96 ± 17.38	128.17 ± 11.61	<0.0001
Weight (kg)	34.45 ± 12.31	29.34 ± 9.41	34.08 ± 11.30	38.50 ± 14.52	47.61 ± 16.74	36.59 ± 7.26	<0.0001
Numbers of BMI measurements	3 (2–3)	4 (3–5)	4 (3–5)	4 (3–5)	4 (3–5)	4 (4–5)	0.0138
SBP (mmHg)	100.25 ± 9.23	97.57 ± 8.44	99.95 ± 9.08	102.88 ± 8.95	107.33 ± 11.55	100.29 ± 7.59	<0.0001
DBP (mmHg)	65.58 ± 7.06	63.87 ± 6.75	65.29 ± 6.98	67.49 ± 6.77	69.94 ± 8.34	66.46 ± 6.30	<0.0001
SBP-z score	−0.71 [−1.41–(−0.04)]	−0.76 [−1.56–(−0.16)]	−0.78 [−1.42–(−0.07)]	−0.54 (−1.25–0.17)	−0.24 (−1.06–0.66)	−0.80 (−1.88–0.10)	<0.0001
DBP-z score	0.15 (−0.35–0.64)	0.09 (−0.36–0.52)	0.08 (−0.38–0.60)	0.25 (−0.16–0.89)	0.34 (−0.20–0.92)	0.09 (−0.64–1.05)	<0.0001
BMI (kg/m^2^)	17.30 ± 2.90	15.10 ± 1.60	17.09 ± 2.30	18.72 ± 2.99	21.99 ± 3.33	22.00 ± 2.52	<0.0001
Overweight	89 (4.75%)	1 (0.27%)	21 (2.00%)	6 (1.88%)	11 (16.18%)	30 (50.85%)	<0.0001
Obesity	69 (3.69%)	1 (0.27%)	18 (1.71%)	19 (5.96%)	31 (45.59%)	20 (33.90%)	<0.0001

*Early adulthood*							
** **Educational level							
** **Primary school and below	382 (20.41%)	81 (21.60%)	230 (21.88%)	56 (17.55%)	11 (16.18%)	4 (6.78%)	0.0721
** **Lower middle school degree	8 (0.43%)	18 (4.80%)	66 (6.28%)	25 (7.84%)	8 (11.76%)	1 (1.69%)	
** **Upper middle school degree	118 (6.30%)	2 (0.53%)	4 (0.38%)	2 (0.63%)	0 (0.00%)	0 (0.00%)	
** **Technical or vocational degree	4 (0.21%)	1 (0.27%)	1 (0.10%)	2 (0.63%)	0 (0.00%)	0 (0.00%)	

** **SBP (mmHg)	111.63 ± 10.57	108.80 ± 10.17	111.75 ± 10.43	113.51 ± 10.91	115.90 ± 10.75	112.41 ± 9.13	<0.0001
** **DBP (mmHg)	73.19 ± 7.89	71.49 ± 7.34	73.24 ± 7.94	74.17 ± 8.15	76.57 ± 8.09	73.88 ± 6.54	<0.0001
** **Hypertension (*n*, %)	162 (8.65%)	17 (4.53%)	99 (9.42%)	35 (10.97%)	6 (8.82%)	5 (8.47%)	0.0265

^*∗*^Age at enrollment for the current study.

**Table 3:** Associations between BMI trajectories and adult hypertension.

	Model 1			Model 2			Model 3		
	OR	95% CI	*P*	OR	95% CI	*P*	OR	95% CI	*P*
Low slow-increasing	1.00	Reference	—	1.00	Reference	—	1.00	Reference	—
Low moderate-increasing	2.39	(1.37–4.17)	**0.0022**	2.66	(1.50–4.72)	**0.0008**	2.48	(1.39–4.42)	**0.0021**
Low rapid-increasing	2.94	(1.57–5.50)	**0.0007**	3.95	(2.05–7.59)	**<0.0001**	3.24	(1.66–6.31)	**0.0005**
Moderate-increasing	2.66	(1.04–6.80)	**0.0411**	4.83	(1.80–12.96)	**0.0018**	3.28	(1.19–9.08)	**0.0222**
Elevated-decreasing	2.18	(0.81–5.82)	0.1198	3.07	(1.10–8.59)	**0.0326**	2.74	(0.98–7.65)	0.0543

OR, odds ratio; 95% CI, 95% confidence interval. Model 1 was unadjusted; Model 2 was adjusted for sex (males, females) and age (in years, continuous) at the last BP measurement and education; and Model 3 was additionally controlled for mean values of SBP and DBP (mmHg, continuous) in childhood, based on Model 2.

## Data Availability

The data used to support the findings of this study were obtained from China Health and Nutrition Survey (https://www.cpc.unc.edu/projects/china/data).

## Abbreviations

BP: Blood pressure
CHNS: China Health and Nutrition Survey
CVD: Cardiovascular disease
BMI: Body mass index
SBP: Systolic blood pressure
DBP: Diastolic blood pressure
OR: Odds ratio
95% CI: 95% confidence interval.

## Supplementary Materials

Supplementary Materials Supplementary Figure 1. Flow chart of optimal model selection for the latent class growth mixture (LCGM) model.
