# Supplementary material for: Body Mass Index Trajectories during 6–18 Years Old and the Risk of Hypertension in Young Adult: A Longitudinal Study in Chinese Population
Source: Int J Hypertens. 2021 Jul 15;2021:6646868. doi: 10.1155/2021/6646868 (PMC8302370; doi:10.1155/2021/6646868)
Supplement: Supplementary Materials — Supplementary Figure 1. Flow chart of optimal model selection for the latent class growth mixture (LCGM) model. [file 6646868.f1.docx]

**
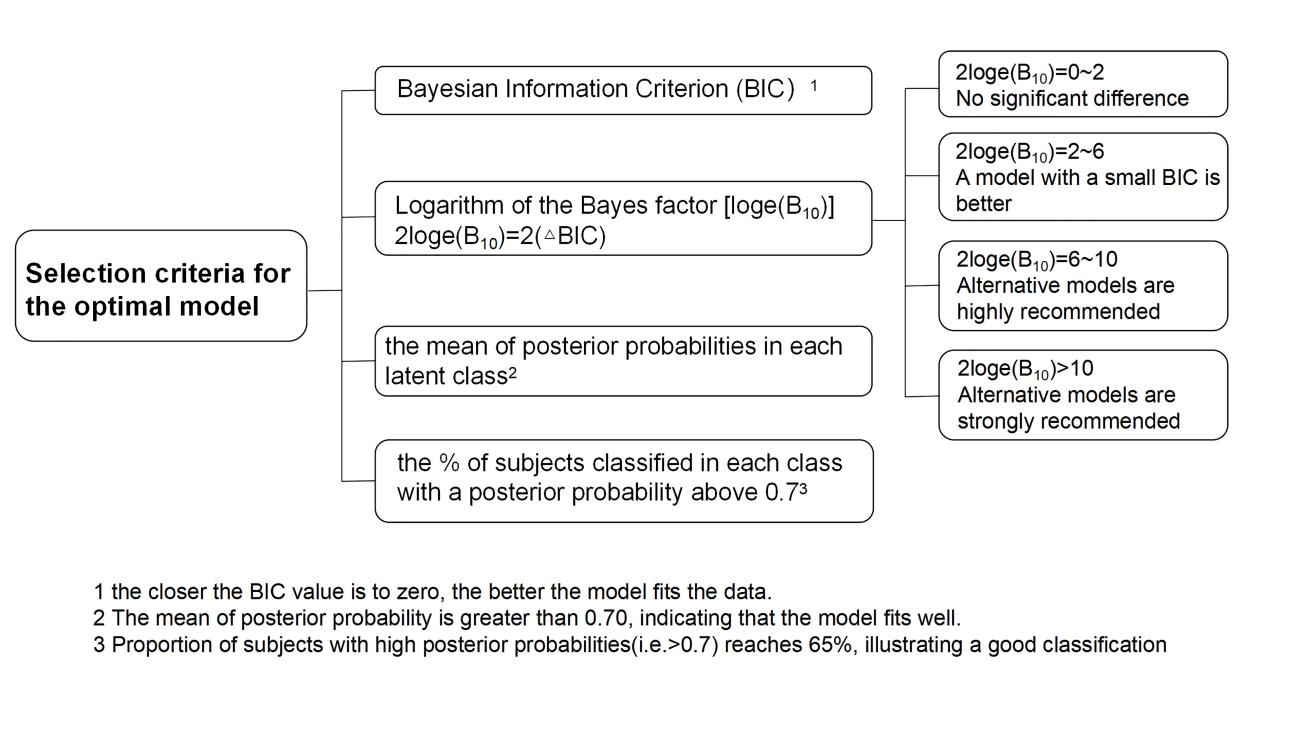
**

**Supplementary Figure 1.** Flow chart of optimal model selection for the latent class growth mixture (LCGM) model.
